# A simple CD4+ T cells to FIB-4 ratio for evaluating prognosis of BCLC-B hepatocellular carcinoma: a retrospective cohort study

**DOI:** 10.1186/s12885-022-09433-3

**Published:** 2022-03-23

**Authors:** Yong Zhao, Ling Xiang Kong, Feng Shi Feng, Jiayin Yang, Guo Wei

**Affiliations:** 1 Department of Liver Surgery and Liver transplantation Laboratory, West China Hospital of Sichuan University, Sichuan Province, Chengdu, China; 2 Department of General Surgery, Chengdu Public Health Clinical Medical Center, Sichuan Province, Chengdu, China

**Keywords:** Human immunodeficiency virus (HIV), Hepatocellular carcinoma (HCC), Barcelona Clinic Liver Cancer (BCLC), Hepatitis B virus (HBV); Platelet (PLT), Alanine aminotransferase (ALT), Aspartate aminotransferase (AST), Aspartate aminotransferase-to-platelet ratio index (APRI), Fibrosis-4 (FIB-4)

## Abstract

**Introduction:**

Immunotherapy has become a new therapy for advanced hepatocellular carcinoma (HCC); however, its treatment results are considerably different. CD4+ T cells (CD4+) are the key to immunotherapy, but patients with HCC that have low CD4+ are rarely observed for clinical evidence. Hepatitis B virus-related HCC is often accompanied by cirrhosis and portal hypertension; therefore, CD4+ tend to be relatively low in number. TACE is the standard treatment for Barcelona Clinic Liver Cancer (BCLC)-B HCC, which may further reduce the number of CD4 + .

**Methods:**

This retrospective cohort study further reduced CD4+ by including patients with human immunodeficiency virus (HIV) to observe the relationship between CD4+ and Chronic hepatitis B virus (CHB) induced HCC. A total of 170 BCLC-B HCC patients (42 HIV+) were included. Univariate and multivariate analyses, and artificial neural networks (ANNs) were used to evaluate the independent risk factors for the two-year survival.

**Results:**

The statistical analysis of the two-year survival rate showed that the main factors influencing survival were liver function and immune indices, including CD4+, platelet, alanine aminotransferase, aspartate aminotransferase, aspartate aminotransferase-to-platelet ratio index, and fibrosis-4 (FIB-4) (*P* < 0.05). Compared with that in other indices, in logistic and ANN multivariate analysis, CD4 + -to-FIB-4 ratio (CD4+/FIB-4) had the highest importance with 0.716 C-statistic and 145.93 cut-off value. In terms of overall survival rate, HIV infection was not a risk factor (*P* = 0.589); however, CD4+/FIB-4 ≤ 145.93 significantly affected patient prognosis (*P* = 0.002).

**Conclusion:**

HIV infection does not affect the prognosis of BCLC-B HCC, but CD4+ have a significant predictive value. CD4+ played a vital role in HCC and this deserves the attention from physicians. Further, the CD4+/FIB-4 is a clinically valuable effective prognostic indicator for these patients.

## Introduction

Immunological checkpoint inhibitor (ICPI) is an new potential therapeutic for advanced hepatocellular carcinoma (HCC), which has been reported to show excellent results in other malignant tumors, such as melanoma, renal cell carcinoma, triple negative breast cancer and non-small cell lung cancer [1]. The main representative drugs that act as ICPI include CTLA-4 and PD-1 inhibitors. CTLA-4 is expressed in activated CD4+ and CD8+ T cells, which can prevent activating effector T cells, and its inhibitor can induce T cell activation, promote the activation and proliferation of effector T cells, and enhance the tumor killing ability [2]. PD-1 is a negative regulatory molecule of T cells. The expression level of PD-1 in CD4+ T cell subsets and CD4+ T cells in patients with chronic viral hepatitis is also substantially different from that in healthy individuals, and its effect is also closely related to CD4+ and CD8+ T cells [3]. However, due to individual heterogeneous advanced HCC, even patients within the same subgroup have been reported to have significantly different outcomes to treatment [4].

Chronic hepatitis B virus (CHB) infection is a high-risk factor for HCC, and responsible for 50–80% of HCC cases worldwide [5]. T-lymphocyte failure is significantly associated with hepatitis B virus (HBV) replication level. Absence of CD4+ T cells can impair CD8+ T cell activity and antibody production, while the inability to mount a virus-specific CD8+ T cell response results in a level of circulating HBV that cannot be cleared by antibodies alone [6, 7]. While some reports suggest that CD4+ T cells may be potential prognostic markers and therapeutic targets for HCC treatment [8], other studies found no correlation between CD4+ T cells and HCC progression [9, 10]. Due to all the above, the relationship between CHB-induced HCC and CD4+ T cells requires clinical evidence.

As cirrhosis mediates the causal pathway to HCC, patients with CHB-induced HCC often have a certain degree of sclerosis and portal hypertension, and their CD4+ T cells levels are often lower than normal. Circulating and liver-infiltrating CD4+ cytotoxic T lymphocytes (CTLs) are significantly increased in patients with HCC at an early disease stage, but decreased in progressive stages [8]. After TACE treatment, the proportion of CD4+ T cells further decreases [11]. Patients with HIV have also low peripheral blood CD4+ T cells counts. Therefore, to better observe the CD4+ T cells in these patients, this study was designed particularly for patients with BCLC-B HCC, including some with HIV. Additionally, liver condition is also the most important factor to be considered in the successful implementation of immunotherapy for patients with advanced HCC. Biopsy is the gold standard for understanding the degree of liver fibrosis or damage; however, it has limitations, including invasiveness, complications and non-dynamic observation of fibrosis/cirrhosis. Currently, multiple non-invasive methods based on inexpensive laboratory tests predict liver fibrosis, including the aspartate aminotransferase-platelet index (APRI) and the fibrosis index based on the four factors (FIB-4). After conducting a meta-analysis and comparing the results with those from previous studies, we believe that these two indicators have high accuracy [12]. This study aimed to provide clinical evidence on the relationship between absolute number of CD4+ T cells and CHB-induced HCC, observe whether the prognosis of HCC is related to HIV, and provide reference clinical evidence and useful index for subsequent immunotherapy grouping in the era of immunotherapy.

## Methods

### Patients

**Fig. 1:**
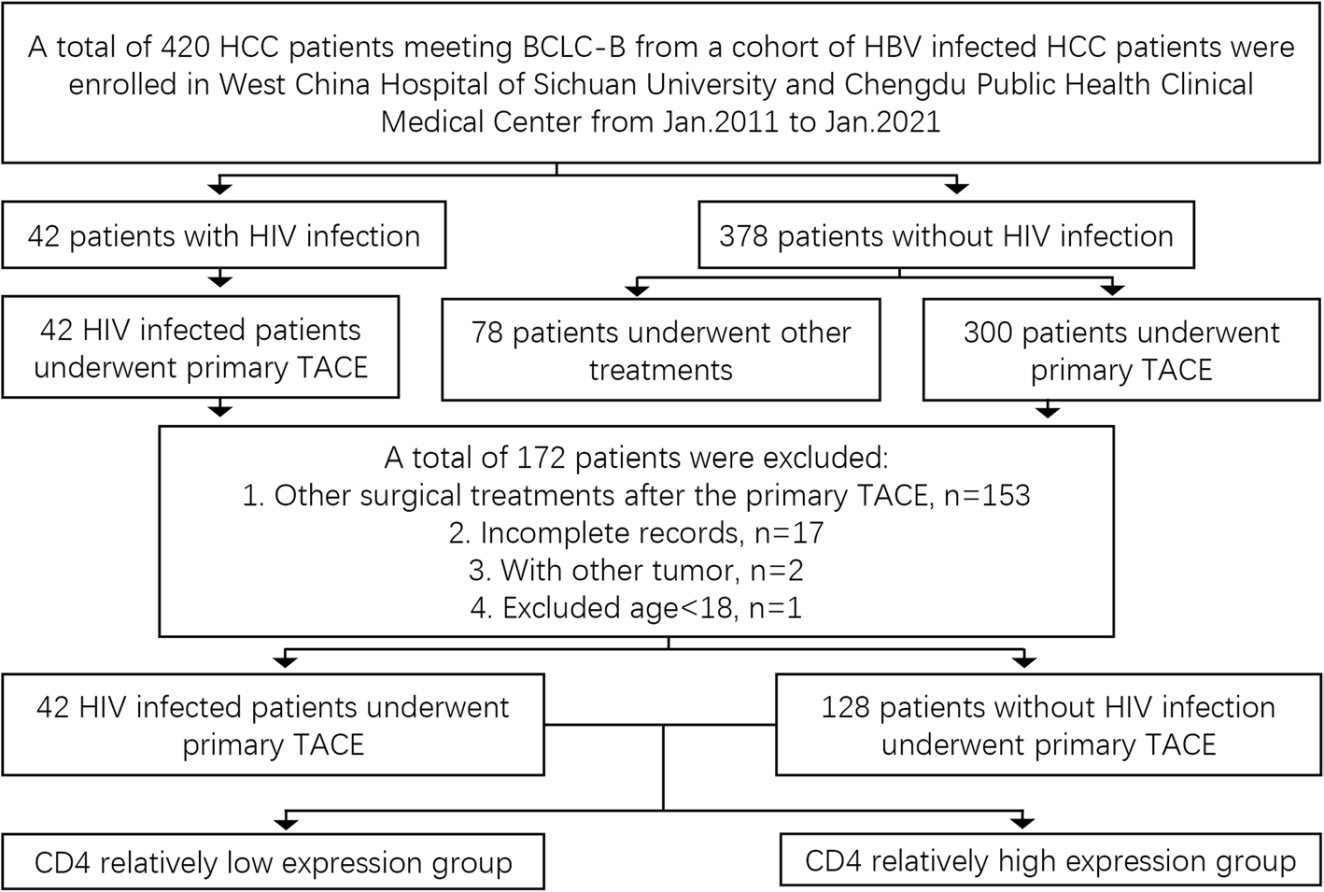
Flow of study participants

Figure 1 details the study design and patient grouping. Only patients were included who were 18 years of age or older. To ensure the consistency of baseline data, none of the enrolled patients received any other therapies before surgery. Written informed consent was obtained from all the patients prior to their surgery, and all of patients were voluntary and altruistic in all cases, and were in accordance with the ethical guidelines of the Declaration of Helsinki. HCC was diagnosed and managed according to the European Association for the Study of the Liver guidelines [13], American Association for the Study of Liver Diseases updated practice guidelines [14], and the Barcelona Clinic of Liver Cancer guidelines [15]. They were monitored until September 2021 or until their death, and their medical records were retrospectively reviewed. The end point was the 2 – year survival rate, which was the median survival time according to the BCLC standard [16], and A cumulative meta-analysis showed that TACE increased the proportions of patients that survived 2 years [17]. The cut-off value of AFP is ≥400 ng / ml, which is mainly based on the current research that HCC patients with serum AFP levels ≥400 ng / ml had a distinctly poor diagnosis with the lowest recurrence free survival rates [18]. The preoperative blood sample acquisition and imaging examination were completed 3 days before TACE. All patients underwent regular review with liver function tests, serum AFP and imaging studies. The first-time postoperative follow-up was 3–4 weeks after TACE. The related imaging examination including general abdominal color doppler ultrasound, contrast-enhanced liver ultrasound, CT or MRI, were performed according to clinical needs every one, two or three months. TACE was repeated at 1–2-month intervals, depending on the tumor burden and response.

### Liver function

Blood was obtained from the peripheral blood of patients who volunteered to participate in our study, then centrifuged at 2000 g for 10 min to obtain the serum. The serum levels of alanine transaminase (ALT), aspartate transaminase (AST), total bilirubin (TB), albumin and prothrombin time (PT) were detected by an automatic biochemical analyzer (Hitachi, Tokyo, Japan).

### Peripheral blood mononuclear cells

PBS-diluted blood samples (1:1) were carefully layered on 2 ml human lymphocyte separation medium (Dakewe, Shenzhen, China). Single-cell suspensions were harvested by gradient centrifugation at 800 g for 30 min. PBMCs were washed twice for further experiments.

### Flow cytometry

Flow cytometry was used to detect CD3+, CD4+ and CD8+ cells. The antibodies added were FITC-anti-CD3 (BD Biosciences), phycoerythrin-anti-CD4 (BD Biosciences) and allophycocyanin-Cy7-anti-CD8 (BD Biosciences), and then incubated at 4 °C for 20 min. Following centrifugation at 200 x g for 5 min at room temperature, the PBMCs were resuspended with PBS containing 1% paraformaldehyde for fixation at room temperature, the result data were acquired by flow cytometry on a FACS Canton II (BD Biosciences) and analyzed by FlowJo software.

### Statistical analysis

R (version 4.0.5) was used to analyze the relevant data. Overall patient survival was estimated by the Kaplan–Meier method, and differences between two groups were determined by log-rank test. Logistic regression and ANN analysis were used to determine the preoperative independent risk factors or protective factors for the two-year survival of patients after TACE. Categorical data were presented as number (per cent) and compared using Pearson chi-Square, Fisher’s exact test. Continuous variables were expressed as the mean value ± SD and analyzed using t-test and repeated measure analysis of variance. *P* < 0.05 was considered to be statistically significant.

## Results

### Baseline demographic disease features

The disease and clinical characteristics of the study population are shown in Table 1. The main differences between patients who did not achieve a 2-year survival and those with relatively good prognosis related to liver function and immune indexes, with ALT, AST, APRI, FIB-4 (all *P* < 0.05). In addition, the absolute number of CD4+ T cells showed a significant statistical difference in immune indices (*P* < 0.05). In the same way, we conducted two classifications according to the median of CD4+ T cells (Table 2). The results show that the average value of Prothrombin time in the high CD4+ T cells group was higher than that in the low CD4+ group (14.78 ± 2.97 vs. 13.84 ± 1.75, *P* = 0.013).

**Table 1:** Baseline demographic and disease features characteristics

Variables	All included patients		
	Death (*n* = 93)	Survival (*n* = 77)	*P*
Non-Liver Function or Immune Related Index			
Age (mean ± SD, years)	50.67 ± 9.33	49.53 ± 12.06	0.501
Male	75 (80.6%)	60 (77.9%)	0.662
BMI (mean ± SD, kg/m2)	22.02 ± 2.07	21.88 ± 1.91	0.651
HB (mean ± SD,g/dl)	127.88 ± 19.63	132.5 ± 19.9	0.131
CRE (mean ± SD,μmol/L)	64.45 ± 16.67	65.27 ± 17.62	0.756
Maximum tumor diameter (mean ± SD, mm)	79.8 ± 22.62	82.97 ± 24.01	0.376
Serum AFP > 400 ng/mL	71 (76.3%)	55 (71.4%)	0.466
Number of tumor ≥2	63 (67.7%)	51 (66.2%)	0.835
Liver Function Related Marker/Index			
Plt (mean ± SD,109/L)	124.41 ± 50.52	137.21 ± 36.34	0.065
ALT (mean ± SD, u/L)	40.89 ± 19.13	49.99 ± 25.91	0.012
AST mean ± SD, u/L)	83 ± 45.94	65.6 ± 39.31	0.010
Alb (mean ± SD,g/L)	39.56 ± 6.37	38.79 ± 5.34	0.404
PT (mean ± SD, s)	14.17 ± 2.21	14.47 ± 2.77	0.446
TB (mean ± SD,μmol/L)	17.93 ± 8.01	17.25 ± 6.62	0.550
Child-Pugh score (mean ± SD)	6.09 ± 1.01	5.94 ± 0.92	0.314
Child-Pugh A	66 (71%)	61 (79.2%)	0.218
Child-Pugh B	27 (29%)	16 (20.8%)	0.218
Child-Pugh C	–	–	–
APRI	1.79 ± 1.1	1.27 ± 0.88	0.001
FIB-4	6.27 ± 4.47	3.84 ± 2.64	0.000
Immune Related Marker/Index			
HIV(+)	24 (25.8%)	18 (23.4%)	0.715
WBC (mean ± SD,109/L)	5.47 ± 1.9	5.67 ± 1.61	0.475
CD3+ T cells	923.47 ± 269.47	997.83 ± 366.96	0.130
CD4+ T cells	426.08 ± 139.34	518.04 ± 242.79	0.004
CD8+ T cells	399.94 ± 140.93	445.37 ± 196.17	0.091
CD4/CD8	1.15 ± 0.59	1.31 ± 0.71	0.129
CD4/APRI	329.17 ± 250.42	646.55 ± 764.37	0.001
CD4/FIB-4	102.23 ± 74.32	216.17 ± 223.32	0.000

*BMI* Body mass index, *CRE* Creatinine, *ALB* Albumin, *TB* Total bilirubin, *INR* International normalized ratio, *MELD* Model end-stage liver disease, *PLT* Platelet, *WBC* White blood cell, *HGB* Hemoglobin, *HBsAg* Hepatitis B surface antigen

**Table 2:** Baseline demographic and disease features characteristics based on CD4

Variables	CD4+ T cells		
	>median (*n* = 85)	≤median (*n* = 85)	*P*
CD4+ T cells	593.3 ± 188.34	342.08 ± 106.84	<0.01
Age (mean ± SD, years)	50.24 ± 9.05	50.07 ± 12.07	0.920
Male	67 (78.8%)	68 (80%)	0.850
BMI (mean ± SD, kg/m2)	21.95 ± 2.12	21.95 ± 1.88	1.000
HB (mean ± SD,g/dl)	131.19 ± 20.65	128.76 ± 19.01	0.426
CRE (mean ± SD,μmol/L)	64.65 ± 21.26	64.98 ± 11.55	0.901
Maximum tumor diameter (mean ± SD, mm)	0.41 ± 0.50	0.34 ± 0.48	0.345
Serum AFP > 400 ng/mL	66 (77.6%)	60 (70.6%)	0.293
Number of tumor ≥2	62 (72.9%)	52 (61.2%)	0.103
Plt (mean ± SD,109/L)	130.32 ± 50.68	130.09 ± 38.79	0.974
ALT (mean ± SD, u/L)	42.25 ± 19.95	47.78 ± 25.22	0.115
AST mean ± SD, u/L)	76.55 ± 49.96	73.68 ± 36.89	0.671
Alb (mean ± SD,g/L)	39.58 ± 6.57	38.84 ± 5.20	0.419
PT (mean ± SD, s)	13.84 ± 1.75	14.78 ± 2.97	0.013
TB (mean ± SD,μmol/L)	23.74 ± 12.37	21.52 ± 10.32	0.206
Child-Pugh score (mean ± SD)	6.09 ± 1	5.94 ± 0.94	0.305
Child-Pugh A	59 (69.4%)	68 (80%)	0.112
Child-Pugh B	26 (30.6%)	17 (20%)	0.112
Child-Pugh C	–	–	–
APRI	1.59 ± 1.14	1.52 ± 0.92	0.658
FIB-4	5.22 ± 3.83	5.11 ± 4.05	0.861

*BMI* Body mass index, *CRE* Creatinine, *ALB* Albumin, *TB* Total bilirubin, *INR* International normalized ratio, *MELD* Model end-stage liver disease, *PLT* Platelet, *WBC* White blood cell, *HGB* Hemoglobin, *HBsAg* Hepatitis B surface antigen

### Risk factor analysis

Based on ROC curve analysis with a 2-year survival rate as outcome index, we calculated the best cut-off value of the statistically significant factors in Table 1, as shown in Table 3. In the univariate analysis, we processed the continuous variables that did not conform to the Gaussian distribution into binary variables. The results from the univariate analysis revealed significant differences in ALT ≤49 U/L, AST > 67 U/L, PLT ≤ 114 × 10^9^ / L, absolute number of CD4+ T cells≤449 /μL, among which the protective factors were ALT, PLT, and CD4 + T cells (Table 4). In terms of indexes, APRI >1.22, FIB-4 > 5.39, CD4 / APRI ≤619.97 and CD4 / FIB-4 ≤ 145.93 also showed significant differences. Only the ratio CD4+/FIB-4 showed a significant difference when included in the multivariate analysis by logistic regression together with other factors or indices (Table 5). The results of the multi-factor analysis using an ANN to analyze the importance of all potential factors are shown in Table 6. Among the factors included, CD4/FIB-4 had the highest importance.

**Table 3:** The AUC value of liver function and immune markers or indexes

Variable	AUC	95% CI	Cut-off	*P* (Area = 0.5)
Child-Pugh score	0.538	0.460–0.615	–	0.385
CD4+ T cells	0.643	0.566–0.715	≤449	0.001
CD4/CD8	0.560	0.482–0.636	–	0.187
AST	0.613	0.535–0.686	>67	0.009
ALT	0.602	0.524–0.676	≤49	0.022
PLT	0.637	0.560–0.709	≤114	0.001
APRI	0.661	0.585–0.732	>1.22	<0.001
FIB-4	0.682	0.607–0.751	>5.39	<0.001
CD4/APRI	0.702	0.627–0.770	≤619.97	<0.001
CD4/FIB-4	0.716	0.642–0.782	≤145.93	<0.001

**Table 4:** The variables in the univariate analysis for the BCLC-B stage

Variables	All included patients (*n* = 1464)	
	Relative risk (95% CI)	*P*
Age (years)	1.010 (0.982–1.039)	0.488
Male	0.534 (0.261–1.091)	0.085
BMI (kg/m^2^)	1.036 (0.890–1.206)	0.649
Child-Pugh score	1.177 (0.858–1.615)	0.312
WBC (10^9^/L)	0.939 (0.791–1.115)	0.472
sCr (μmol/L)	0.997 (0.980–1.015)	0.754
Alb (g/L)	1.023 (0.971–1.077)	0.402
PT (s)	0.953 (0.843–1.078)	0.444
TB (μmol/L) >2ULN	1.954 (0.858–4.451)	0.111
HB (g/dl) <100 g/L	1.229 (0.411–3.669)	0.712
ALT (u/L) ≤49	0.398 (0.212–0.748)	0.004
AST (u/L) >67	2.246 (1.213–4.160)	0.010
Plt (10^9^/L) ≤114	0.389 (0.207–0.732)	0.003
Serum AFP (ng/mL) > 400	1.291 (0.649–2.568)	0.467
Number of tumor ≥2	1.071 (0.563–2.034)	0.835
Maximum tumor diameter (mm) >80	0.816 (0.438–1.521)	0.523
HIV	0.877 (0.434–1.772)	0.715
CD3+ T cells (/μL)	0.999 (0.998–1.000)	0.134
CD4+ T cells (/μL) ≤449	0.326 (0.174–0.611)	<0.001
CD8+ T cells (/μL)	0.998 (0.997–1.000)	0.084
CD4+/CD8+	0.676 (0.403–1.135)	0.138
APRI >1.22	2.872 (1.537–5.366)	0.001
FIB-4 > 5.39	3.404 (1.697–6.829)	0.001
CD4/APRI ≤619.97	0.108 (0.420–0.278)	<0.001
CD4/FIB-4 ≤ 145.93	0.203 (0.103–0.399)	<0.001

*BMI* Body mass index, *HBsAg* Hepatitis B surface antigen, *TB* Total bilirubin, *AFP* Alpha-fetoprotein, *PT* Prothrombin time, *WBC* White blood cell, *sCr* Serum creatinine, *HB* Hemoglobin, *Plt* Platelet, *Alb* Albumin

**Table 5:** Independent variables in the multivariate analysis for 2-year survival

Variables	Logistic regression	
	Relative risk (95% CI)	*P*
Child-Pugh score	0.909 (0.637–1.297)	0.600
CD4/APRI	1.000 (0.998–1.002)	0.666
CD4/FIB-4	0.993 (0.987–0.998)	0.012
AST (u/L)	1.000 (0.991–1.009)	0.970
Serum AFP (ng/mL) > 400	1.283 (0.601–2.739)	0.519
Maximum tumor diameter (mm)	0.996 (0.982–1.011)	0.622

**Table 6:** Independent variables in the ANN analysis for 2-year survival

Variables	Importance	Normalized Importance
CD4+/FIB-4	0.159	100.0%
CD4+/CD8+	0.130	81.8%
CD4+/APRI	0.095	60.0%
CD4+ T cells (/μL)	0.082	51.4%
FIB-4	0.053	33.1%
Plt (10^9^/L)	0.052	32.7%
ALT (u/L)	0.051	32.3%
CD8+ T cells (/μL)	0.048	30.1%
CD3+ T cells (/μL)	0.044	27.9%
APRI	0.040	25.4%
PT (s)	0.039	24.7%
AST (u/L)	0.035	22.4%
HIV	0.035	22.3%
Child-Pugh score	0.032	19.9%
Serum AFP (ng/mL) > 400	0.029	18.1%
Number of tumor >3	0.027	17.0%
TB (μmol/L)	0.027	16.7%
Maximum tumor diameter (mm)	0.024	14.9%

### Patient survival

**Fig. 2:**
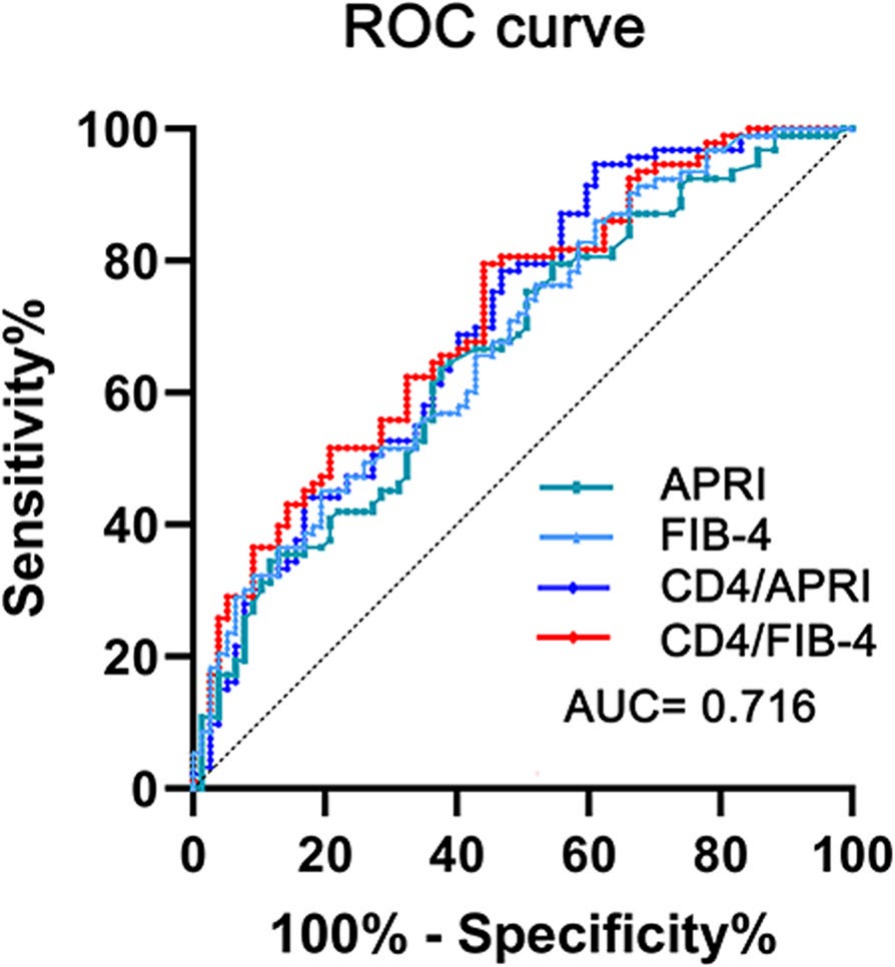
ROC curve of APRI, FIB-4, CD4/APRI, and CD4/FIB-4. AUC of ROC curves corresponding to CD4/FIB-4 ratio is 0.716. Other AUC data results are shown in Table 2

**Fig. 3:**
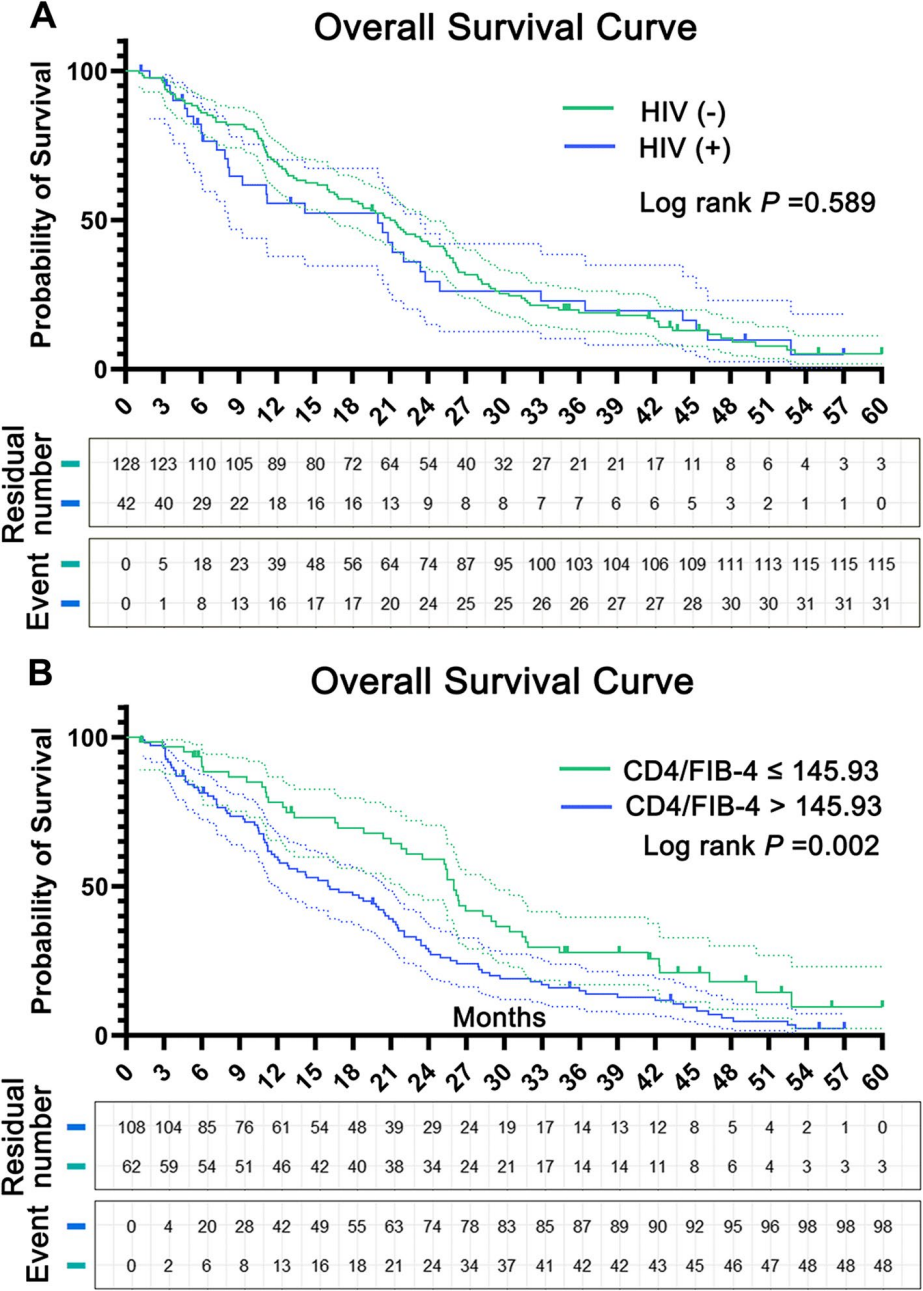
Overall survival. **A** Based on HIV overall survival rate. **B** Based on CD4/FIB-4 overall survival rate

Based on whether patients had an HIV infection and a CD4/FIB-4 ratio ≤ 145.93, we drew the overall survival curve corresponding to the above cut-off value as shown in Figs. 2 and 3. The results showed that the median survival time of the HIV group was 20.03 ± 6.31 months (95% CI 7.67–32.40), and that of the non-HIV group was 21.00 ± 1.81 (95% CI 17.45–24.55). There was no significant difference in the overall survival rate between the two groups (*P* = 0.589). According to CD4/FIB-4 rate ≤ 145.93 grouping, we found a significantly different (*P* = 0.002) median survival time between the high level [26.00 ± 0.56 (24.91 ± 27.09)], and the low level [16.00 ± 2.75 95% CI (10.61 ± 21.39)] groups.

## Discussion

Our study results showed that the prognosis in patients with advanced HCC was closely related to the absolute number of CD4+ T cells. In contrast to previous studies, our study specifically focused on BCLC-B stage HCC, which is very frequent in many countries where the early diagnosis rate of HCC is not high [19]. In addition, the population included in previous studies often lacked a population with a significant decline in the number of CD4+ T cells, which was usually within the normal range. In this retrospective cohort study, we followed a study cohort with the lowest number of CD4+ T cells (57) and the 25% quartile of CD4 (only 387.75) over the last 10 years. By including an HIV-infected population in a special cohort study, we could observe these patients more accurately. We found that CD4+ T cells, rather than HIV infection, had a significant impact on the prognosis of patients with BCLC-B HCC. Through comprehensive statistical analysis, CD4/FIB-4 was proved to be a very effective independent predictor for the prognosis of patients with HCC with a C-statistic of 0.716. This predictor index is composed of the most common clinical test values, and its calculation is simple, and can serve as a clinical reference for regions lacking appropriate laboratory testing conditions and/or clinical data support for in-depth laboratory research.

CD4+ T cells are differently involved in tumor occurrence and development, but mainly through immunosuppression, by Foxp3+ Treg cells and cytotoxic T lymphocytes (Tc), memory T lymphocytes, and T helper lymphocytes (Th) [20, 21]. According to the tumor immune evasion theory, cancer patients may drift between Th1/Th2 and Tc1/Tc2, hence hindering anti-tumor immune function in a variety of malignant tumors [22, 23]. Circulating and liver infiltrating CD4+ CTLs were significantly increased in patients with HCC during early disease stages but decreased in progressive stages. Some studies have revealed that inducing apoptosis of CD4+ T cells can promote HCC development, while rescuing apoptosis of CD4+ T lymphocytes can prevent HCC development [24, 25]. In general, the CD4+/CD8+ ratio is decreased, leading to increased immune tolerance and immunosuppression [26]. An association between CD4/CD8 ratio < 0.4 and prominent T cell activation and senescence was reported in patients with CD4+ T cell counts <500/mm^3^ [27]. However, several studies have reported no correlation between CD4+ T cell levels and HCC progression [9–11]. Although positively correlated with elevated AFP and poor tumor differentiation, CD4+ tumor infiltrating lymphocytes (TILs) are associated with neither overall survival nor disease-free survival [8, 10]. Overall, the detailed mechanisms by which immune cells predict prognosis remains unclear in HCC. A common feature in these studies is that the number of CD4 + T cells is almost at the normal level, and control of patients with low CD4+ number is lacking. Through the selection of the included patients, we successfully observed the cohort of people with low number of CD4+ T cells, and CD4+ T cells had a considerable impact on the prognosis of patients with advanced HCC.

We are currently experiencing the era of tumor immunotherapy; however, ICPI shows that the prognosis of liver cancer treatment is very different. Elucidating the reasons that cause these differences is one of the most important directions of current research. Sorafenib and TACE are the standard treatment for patients with advanced HCC. After sorafenib treatment, the expression and absolute number of PD-1 positive T cells and T regulatory cells are decreased, but those of bone marrow-derived suppressor cells do not change, while the number of PD-1 positive T cells in circulation are decreased significantly. Compared with patients without significant decline, the overall survival is significantly improved. The improvement in overall survival is also significantly better in patients with higher baseline PD-1 positive T cell levels than in those with lower baseline levels [28]. In contrast, at present, there are few reports on immune cells and the results of TACE treatment-related patients. A latest study shows that TACE not only further reduced the CD4+ or CD4+/CD8+ ratio, but also significantly reduced the mRNA expression level of PD1 [11]. However, due to the inclusion of population, the study did not observe a significant impact on CD4+ T cells. Our study demonstrated that CD4+ T cells were positively correlated with the prognosis of patients with liver cancer. Focusing on changes of CD4+ T cells may provide an important research direction for the research of ICPI treatment.

A recent study based on 35,659 HIV infected people has found that higher HIV RNA and longer duration of HIV infection increases HCC risk independently of traditional HCC risk factors, but not the CD4+ T cell number, which is the strongest evidence to date to support the contribution of HIV viremia to HCC risk in this group [29]. However, whether HIV or CD4+ T cells increase the risk of HCC is still controversial [30–32]. Since the introduction of HAART, patients with HIV have a life expectancy comparable to that of the general population [33, 34]. Liver disease has now become one of the leading causes of hospitalization and death in patients with HIV, with HCC being the main cause [35–37]. Moreover, HIV-infected individuals have a four-fold higher risk of suffering with HCC than uninfected individuals [38]. However, whether HIV combined with HBV may increase the risk of HCC is still controversial [30–32]. At present, the largest study reported that this may be related to absolute number of CD4+ T cells rather than the HIV infection [29]. However, other studies reported that neither early- (≤2 years) nor long-lasting (>2 years) HIV suppression decreased HCC risk [39–42]. As for HCC prognosis, in HIV-infected patients, the 5-year survival rate is generally considered worse for non-infected patients [43]. Gelu-Simeon *et al.* performed the largest multicenter study so far, showing that the 1-and 2-year survival rates of HIV-infected patients are significantly worse than those of non-infected patients [44]. In contrast to the above findings, our data show that HIV infection is not an independent risk factor for HCC prognosis in patients with BCLC-B, and the number of CD4+ T cells has a more significant predictive effect. This may be because the patients with HCC that were included in previous studies were at both early (BCLC-0 / A) and advanced disease stages. Advanced HCC is usually accompanied by complications, such as liver cirrhosis and portal hypertension. These factors exacerbate the decrease in the number of peripheral CD4+ T cells [8]. There are big differences in peripheral blood CD4+ T cells levels between these periods. Therefore, cohort studies that continuously monitor CD4+ T cells levels or control studies that clearly distinguish patients with early or advanced HCC are needed to study the high/low CD4 expression levels at different stages of HCC.

At present, the main prognostic risk models for HCC caused by CHB are CU-HCC, GAG-HCC, PAGE-B, mPAGE-B, REACH-B, and mREACH-B [45]. As for HIV-related HCC, the largest single-arm multicenter study of 387 HIV-infected patients with HCC showed that the albumin-bilirubin grade highlights the interplay between liver reserve and immune dysfunction as a potential prognostic factor for the survival of patients with HIV+ HCC [46]. The prognostic risk factors in these models can be summarized as basic information, such as patient age and sex, tumor characteristics and liver function, and prognostic risk factors in published guidelines, consistent with these risk factors [47–49]. Although the predicted efficiency of the C-statistic value for these models or indicators in CHB HCC varies between 0.582–0.785 [50], the latter differ from robust molecular biomarkers for predicting HCC prognosis. These factors originate from strong evidence, are common and cheap, as is the proposed new CD4/FIB-4 index. However, it also differs from previous guidelines and classical models, in that it emphasizes the role of immune cells in HCC development. In our results, the FIB-4 index was a more accurate and noninvasive method for evaluating the degree of liver reserve function than others, which was consistent with a previously published meta-analysis of the noninvasive liver fibrosis index [12]. As for other indicators, such as the AFP, Child–Pugh score, and tumor size, which are usually closely related to the prognosis of CHB-related HCC, we observed no significant difference in them, which may be mainly because the BCLC-B stage has limited tumor characteristics and some indicators of liver function. Based on the results of this targeted cohort study, we believe that clinicians should pay more attention to the prognostic impact of immune cells on CHB BCLC-B HCC.

### Prospects and limitations

The retrospective design should be acknowledged as a limitation of our study, while the single-center data source limits its scope. Whether our results can be generalized requires further multicenter verification. There were 170 patients with CHB undergoing TACE in the BCLC-B stage, while only 42 HIV-infected patients were included in the analysis; however, to our knowledge, this study is the largest case-control study of survival outcomes of BCLC-B stage patients with HIV who underwent TACE.

## Conclusion

Our study shows that the CD4+ T cells number is also significantly correlated with the prognosis of patients with BCLC-B stage, making the immune status of patients with advanced HCC worthy of clinical attention. In our study, liver function and absolute number of CD4+ T cells were the two main aspects affecting patient prognosis, which gradually deteriorate with the aggravation of CHB HCC. Therefore, we suggest that patients with BCLC-B HCC should be treated with TACE as soon as possible. HBV and HIV infections are mainly found in underdeveloped areas, where clinicians can only detect basic common clinical indicators. The CD4/FIB-4 prediction index comprises the most common clinical test values and is easy to calculate. Thus, it can serve as a clinical reference for areas lacking laboratory testing conditions and as clinical data support for future laboratory research. This information may facilitate the development of novel strategies for the early prevention and immunotherapy for patients with HCC by targeting the CD4+ T cells.

## Data Availability

All related data in this study are available from the corresponding author on reasonable request.
